# The pathway ontology – updates and applications

**DOI:** 10.1186/2041-1480-5-7

**Published:** 2014-02-05

**Authors:** Victoria Petri, Pushkala Jayaraman, Marek Tutaj, G Thomas Hayman, Jennifer R Smith, Jeff De Pons, Stanley JF Laulederkind, Timothy F Lowry, Rajni Nigam, Shur-Jen Wang, Mary Shimoyama, Melinda R Dwinell, Diane H Munzenmaier, Elizabeth A Worthey, Howard J Jacob

**Affiliations:** 1 Human and Molecular Genetics Center, Medical College of Wisconsin, Milwaukee, WI, USA; 2 Department of Physiology, Medical College of Wisconsin, Milwaukee, WI, USA; 3 Department of Pediatrics, Medical College of Wisconsin, Milwaukee, WI, USA; 4 Department of Surgery, Medical College of Wisconsin, Milwaukee, WI, USA

**Keywords:** Biological pathway, Ontology, Pipeline, Pathway annotations, Pathway diagrams

## Abstract

**Background:**

The Pathway Ontology (PW) developed at the Rat Genome Database (RGD), covers all types of biological pathways, including altered and disease pathways and captures the relationships between them within the hierarchical structure of a directed acyclic graph. The ontology allows for the standardized annotation of rat, and of human and mouse genes to pathway terms. It also constitutes a vehicle for easy navigation between gene and ontology report pages, between reports and interactive pathway diagrams, between pathways directly connected within a diagram and between those that are globally related in pathway suites and suite networks. Surveys of the literature and the development of the Pathway and Disease Portals are important sources for the ongoing development of the ontology. User requests and mapping of pathways in other databases to terms in the ontology further contribute to increasing its content. Recently built automated pipelines use the mapped terms to make available the annotations generated by other groups.

**Results:**

The two released pipelines – the Pathway Interaction Database (PID) Annotation Import Pipeline and the Kyoto Encyclopedia of Genes and Genomes (KEGG) Annotation Import Pipeline, make available over 7,400 and 31,000 pathway gene annotations, respectively. Building the PID pipeline lead to the addition of new terms within the signaling node, also augmented by the release of the RGD “Immune and Inflammatory Disease Portal” at that time. Building the KEGG pipeline lead to a substantial increase in the number of disease pathway terms, such as those within the ‘infectious disease pathway’ parent term category. The ‘drug pathway’ node has also seen increases in the number of terms as well as a restructuring of the node. Literature surveys, disease portal deployments and user requests have contributed and continue to contribute additional new terms across the ontology. Since first presented, the content of PW has increased by over 75%.

**Conclusions:**

Ongoing development of the Pathway Ontology and the implementation of pipelines promote an enriched provision of pathway data. The ontology is freely available for download and use from the RGD ftp site at ftp://rgd.mcw.edu/pub/ontology/pathway/ or from the National Center for Biomedical Ontology (NCBO) BioPortal website at http://bioportal.bioontology.org/ontologies/PW.

## Background

### Introduction

The Pathway Ontology (PW) originated and is being developed at the Rat Genome Database (RGD) [1]. Its goal is to cover any type of biological pathway, including altered and disease pathways, and to capture the relationships between them within the hierarchical structure of a controlled vocabulary or ontology. The building of biological ontologies as directed acyclic graphs (DAG) and the use of structured or controlled vocabularies was first advanced and implemented by the Gene Ontology (GO) project [2,3]. Many bio-ontologies have been developed since [4], as witnessed by the ever-growing number submitted to and made available at the National Center for Biomedical Ontology (NCBO) BioPortal [5,6]. Several ontologies, including the Pathway Ontology, are being developed at RGD ([7], in the “Biomedical Ontologies” thematic series of the Journal of Biomedical Semantics). Within the structure of a DAG, terms have defined relationships to one another and a particular term can have more than one parent. This means that there can be more than one path in the ontology tree from a broader, more general parent term to a more specialized child term. Within the tree structure, terms are nodes whose names designate the class(es) they represent and which are connected by edges that represent the relationship(s) between them. In PW, a node is the network/pathway class it stands for, and its features and aspects are captured in the definition. A pathway is a set of inter-connected reactions and interactions whose delineation and scope are used as a model for exploring and studying, describing and understanding the working of and relationships between biomolecules within a context. The categories or types of pathways are conceptualized and referenced in the scientific literature and represented in pathway databases such as the Kyoto Encyclopedia of Genes and Genomes (KEGG), the Pharmacogenomics Knowledge Base (PharmGKB), the Small Molecule Pathway Database (SMPDB) and WikiPathways, among others [8-11].

### The pathway ontology structure

**Figure 1:**
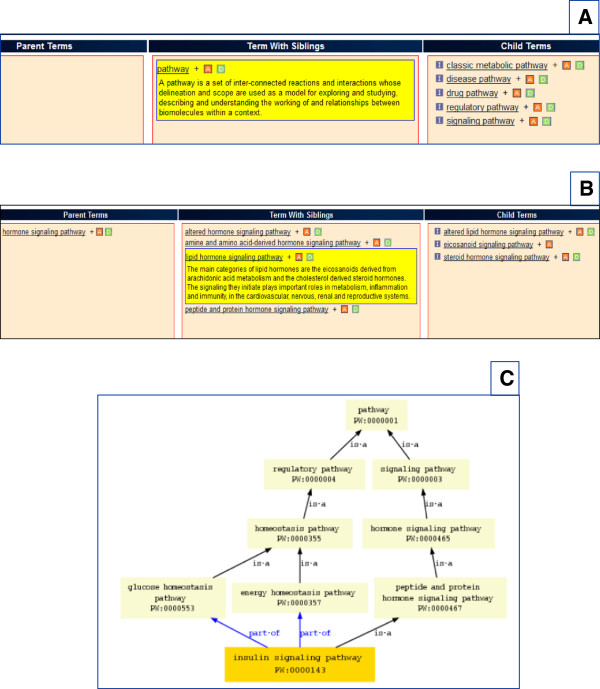
**The pathway ontology main nodes and positions of selected terms. A.** The five nodes of the Pathway Ontology. **B.** The term ‘lipid hormone signaling pathway’ in the ontology showing the parent, siblings and children terms. **C.** The term ‘insulin signaling pathway’ in the ontology showing the position of the term within the tree. ‘Insulin signaling pathway’ is in a part_of relationship to the ‘glucose’ and ‘energy homeostasis pathway’ terms within the regulatory node and in an is_a relationship to ‘peptide and protein hormone signaling pathway’ term within the signaling node.

The first of the main five nodes of the ontology, the metabolic node, contains networks/pathways that stand for/represent the set of reactions underlying the transformation of compounds. The set of reactions/interactions underlying the coordinated responses that maintain the cellular/tissue and/or organ/organismal status quo and homeostasis are placed under the regulatory node. The set of reactions/interactions initiated or triggered by a binding/molecular interaction/conformational change event are found under the signaling node. The set or sets of interactions where one or more are deviant and represent the system’s perturbation(s) fall under the disease node. Finally, the set or sets of reactions/interactions representing the system’s response to and handling of treatment(s) geared towards dealing with those perturbation(s) are housed in the drug node. Thus, the main nodes of the Pathway Ontology are: metabolic, regulatory, signaling, disease and drug pathway (Figure 1A). Two types of relationships are being used in the ontology: “is_a” and “part_of”. For instance, insulin and glucagon are peptide hormones whose signaling - ‘insulin signaling pathway’ and ‘glucagon signaling pathway’, are children terms in an ‘is-a’ relationship to the parent term ‘peptide and protein hormone signaling pathway’. The two signaling pathways which are initiated in response to high levels of circulating glucose – ‘insulin signaling pathway’, or low – ‘glucagon signaling pathway’, and whose engagement of intracellular cascades aims at restoring the normal physiological levels of glucose, are also in a “part-of” relationship to the ‘glucose homeostasis pathway’ term, along with other pertinent terms. Insulin also plays important roles in energy homeostasis. In the brain, insulin (and leptin) act to increase the expression of appetite-decreasing *Pomc* while decreasing the expression of appetite-stimulating *Agrp* genes. The ‘peptide and protein hormone signaling pathway’ term is in turn a child of the more general term ‘hormone signaling pathway’, as other classes of compounds with very different physico-chemical properties can also act as hormones. For instance, the steroid hormones and the eicosanoids which, as the names suggest, are hormones, are lipid molecules. The signaling pathways they initiate are children of the ‘lipid hormone signaling pathway’ term which in turn, is a sibling of ‘peptide and protein hormone signaling pathway’ and child of ‘hormone signaling pathway’ terms (Figure 1B). The nodes are not disjoint and a given pathway class can be the child of terms residing in different nodes, as the examples of insulin and glucagon signaling above show. The ‘peptide and protein hormone signaling pathway’ and the ‘glucose homeostasis pathway’ are both parents of the signaling pathways of insulin and glucagon, albeit with different relationships to their children; the two parent terms are within the signaling and regulatory nodes, respectively. The ‘energy homeostasis pathway’ term is also a parent of insulin signaling and like glucose homeostasis, it is within the regulatory node (Figure 1C).

The “pathway” and the “process” concepts, although at times interchangeably used, are distinct. A pathway conveys the idea of a set of interacting molecules, of the reactions and interactions underlying its functioning. A process on the other hand, conveys the idea of the end result, the conclusion of a plan of action, whether the consequence of the combined work that the set of reactions and interactions produces, in the case of a simpler one, or in the case of a more complex one, the combined work of pathways that contribute to or in some fashion modulate the end result. At the same time, a given pathway can participate in and/or regulate several processes [12]. In the Biological Process (BP) ontology of GO there are metabolic and other process terms that map to KEGG pathways and to terms in PW. For instance, the formation of a fatty acid molecule is the ‘fatty acid biosynthetic process’ term in GO; it is the ‘fatty acid biosynthetic pathway’ term and the ‘fatty acid biosynthesis’ entry in PW and at KEGG, respectively. While the phrasing is similar in GO, PW and KEGG, the term represents a process in GO, a pathway in PW and the KEGG database. KEGG is a primary source for metabolic pathways and projects such as databases and ontologies that in some fashion represent metabolism are going to exhibit a sharing, or an overlapping of terms/entries naming, but not an overlapping of concepts and/or contexts. Likewise, there are signaling pathway terms in BP that relate to similar terms in the signaling pathway node of PW and map to entries in pathway databases such as KEGG and others. However, the positions of and relationships between such terms are different, as are the perspectives of the two ontologies.

### Disease and altered pathways

The provision of terms for the altered versions of pathways and the representation of disease pathways and diagrams as collections of altered pathways are unique to PW and its use at RGD. An altered pathway is one where defects in one or several components of the pathway affect its normal functioning with potential implications for a diseased phenotype. The severity of an altered pathway or the convergence of several altered pathways can overcome the ability of the system to adjust and is manifested in the diseased state. Viewing diseases from a network- rather than a gene-centric perspective, from the systems level of pathway cross-talk and alterations within, is an approach increasingly being considered [13-15].

**Figure 2:**
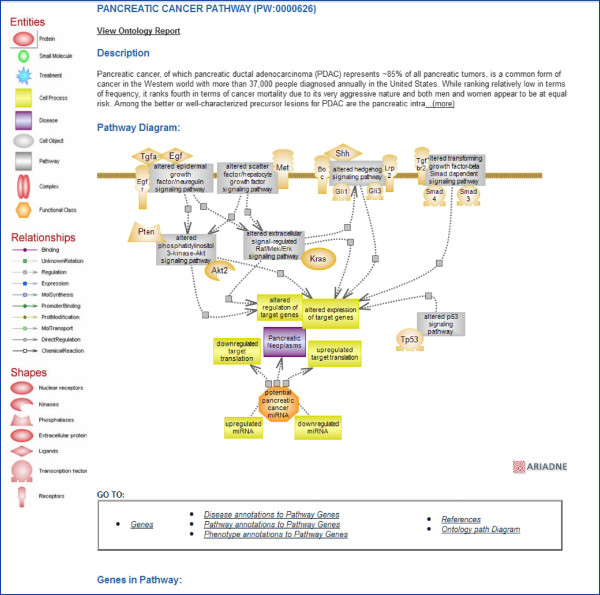
**Pancreatic cancer pathway diagram.** The interactive pathway diagram page for the ‘pancreatic cancer pathway’. The altered pathways associated with the condition are shown as gray rectangles that link to the ontology report(s) for the those terms. Culprit genes within the pathways are shown color-coded (default is red). The icon for the microRNAs (miRNA) with potential roles in pancreatic cancer links to a page where several down- and up-regulated miRNAs are shown with some targets listed and with links to their report pages in RGD and the microRNA database (MiRBase). The icon for the condition links to the Cancer Disease Portal in RGD.

As an example, a large-scale study carried out on a number of pancreatic tumors identified several sets of genes that were altered in the majority of tumors. Of these, many were associated with core signaling pathways and altered in 67% to 100% of tumors [16]. Perhaps not surprisingly, these are pathways important for growth and proliferation and in some cases, also known to be oncogenic (Figure 2). What may be intriguing is the relatively large number of altered pathways and one is tempted to wonder/speculate whether it is this number and the combinations that result from it, that overcome the ability of the system to adjust and/or recover and render the condition intractable. The pancreatic cancer pathway diagram presents the main pathways altered in the condition with the culprit genes shown color coded. Additional links to a list of miRNAs (microRNAs) aberrantly expressed in pancreatic tumors and to the Cancer Portal at RGD are provided (see Figure 2).

### Pathway annotations, interactive pathway diagrams, pathway suites and suite networks

**Figure 3:**
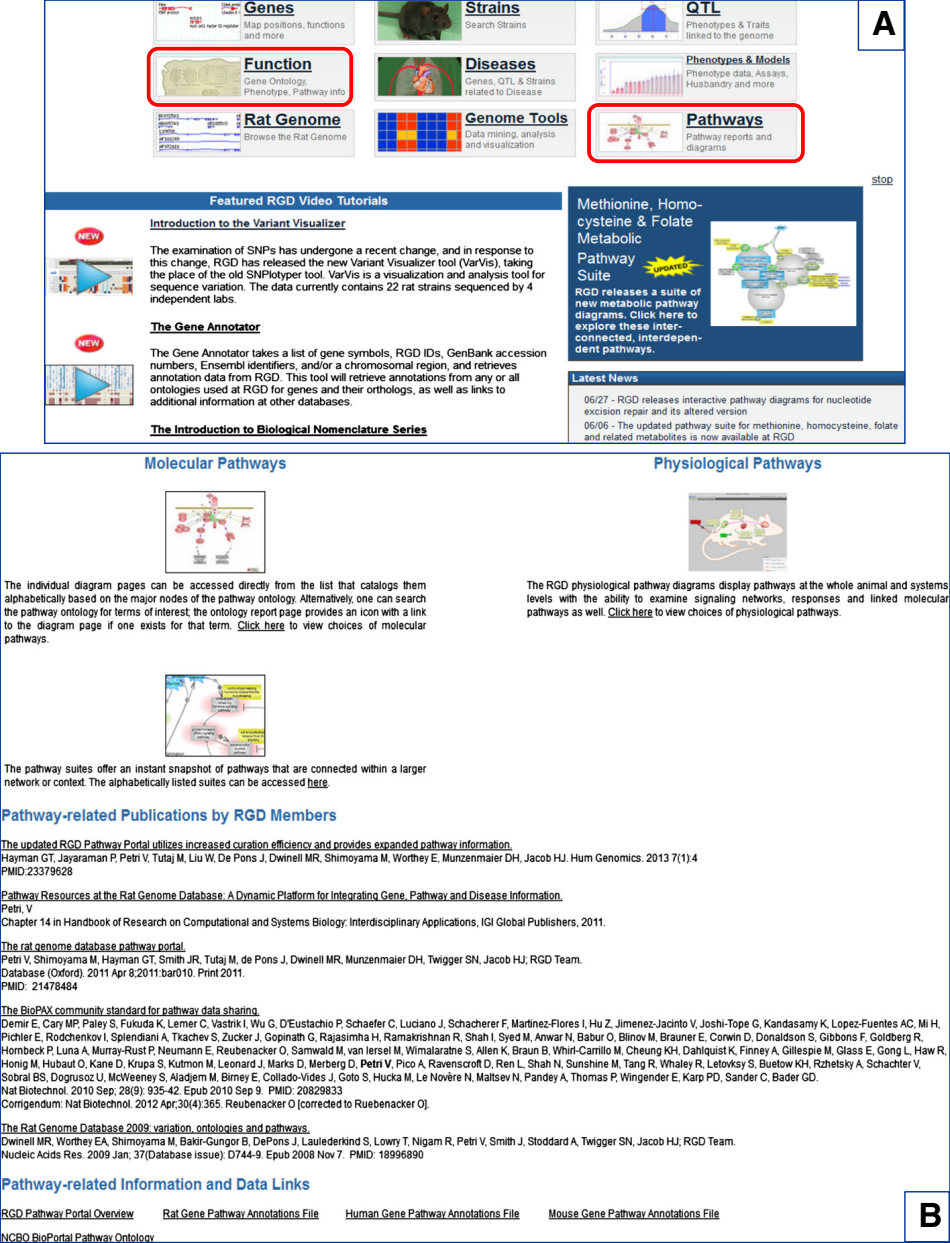
**Pathway portal data access. A.** Rat Genome Database homepage with the main entry points to its content; the “Pathways” and “Function” entry points described in the text, are circled. **B.** Accessing the “Pathways” entry point and entries within.

The use of the ontology allows for the standardized annotation of rat, human and mouse genes to pathway terms. Generally, annotations are made for the term rather than on a gene-by-gene basis; thus, what is being targeted for annotation is the pathway itself – like the ontology the overall pathway curation process is network-centered [12,17]. Importantly, the ontology provides the navigational means to access pathway annotations, interactive pathway diagrams, pathway suites and suite networks as well as a variety of tools, from many entry points. A pathway suite is a collection of pathways that revolves around a common concept or is globally related. If two (or more) pathway suites relate in some fashion, they constitute a suite network. For instance, the ‘Glucose Homeostasis Pathway Suite Network’ brings together the suite dedicated to the various metabolic pathways involving glucose and the one dedicated to the contributing signaling and regulatory pathways. Together, the pathway ontology, the pathway annotations and the graphical representations of pathways, constitute the elements of the Pathway Portal [12,17,18], an important project at the Rat Genome Database [19,20]. Pathway, along with disease, phenotype and biological process, are the major concepts around which the Disease Portals are built and are entry points to access the data they contain. The Disease and Pathway Portals can be accessed from the main homepage of RGD (Figure 3A). The “Pathways” entry point leads to the Molecular Pathways link which houses the collection of interactive pathway diagrams and suites that RGD publishes. This entry point also provides access to pathway related publications by members of RGD as well as other information and data links (Figure 3B).

An ontology search, accessed through the “Function” entry point (see Figure 3A), brings up all the ontologies that have terms which contain the keyword(s) used. Selection of an ontology will show the terms containing the keyword(s) with the option to search the tree or view the annotations. Selecting the branch icon to the left of a term brings up a browser result showing the parent, siblings and children of the term. The browser has been developed at RGD and recently updated to indicate whether interactive pathway diagrams are available or not for terms and/or their children in the form of a boxed “D” of darker or paler green color, respectively (see Figure 1A-B). Any dark green “D” box links to that interactive diagram page. In addition, if the searched term has a diagram, a small icon will be shown in the term entry, to the right of the term description; it will also link to the diagram page. [The boxed “A” in Figure 1A-B denotes the presence of annotations]. Selecting a term brings up an ontology report page with the GViewer tool – a genome-wide view of rat chromosomes with genes annotated to the term, a tabular list of genes annotated to the term by species with links to respective gene report pages and a diagram showing the paths to the root term in the ontology tree. If there is an interactive pathway diagram for the chosen term, an icon is present at the top of the page to the right of the diagram and it links to the pathway diagram page.

**Figure 4:**
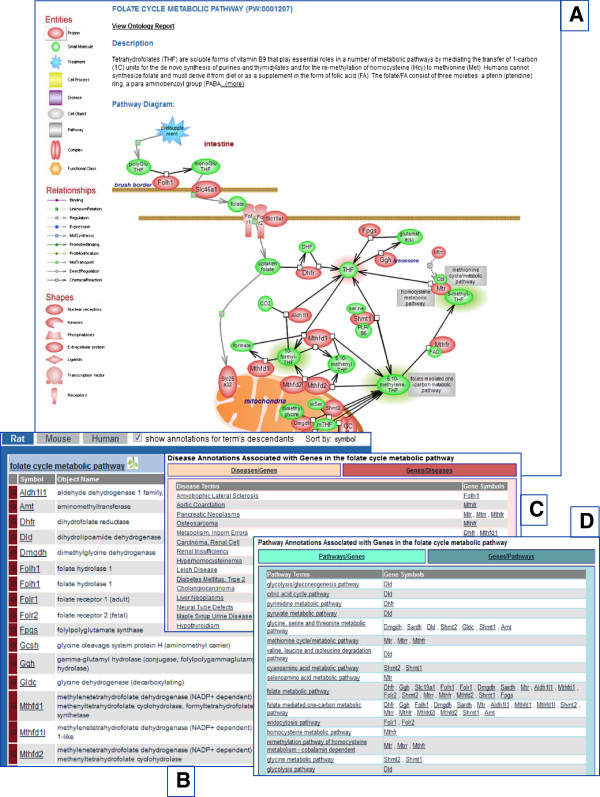
**The anatomy of an interactive pathway diagram page. A.** The top of the page shows the beginning of the description with the option of viewing the whole text and the diagram below it. **B.** The genes in the pathway are shown by species in a tabular form with various link options. **C.** Genes in the pathway that have disease annotations are shown in a table that can be toggled between diseases, alphabetically listed, with the associated genes shown to the right (default), and genes, alphabetically listed, with the associated diseases shown to the right. **D.** Genes in the pathway that have annotations to other pathways are shown in a table that can be toggled between pathways, alphabetically listed, with the associated genes shown to the right (default), and genes, alphabetically listed, with the associated pathways shown to the right. The last section of the diagram page has the reference list as well as a view of the ontology tree (not shown).

Every diagram page consists of several sections. The first provides an in-depth, expandable description of the pathway and the diagram itself whose objects link to their report pages in RGD (genes, chemicals, pathways) or other websites. Beneath that is a tabular list of annotated genes by species with each entry linking to its report page and other links. As applicable, the altered version of the pathway and additional elements in the diagram can also be found in this section. The next section contains tabular lists of genes in the pathway that have been annotated to disease, other pathway and phenotype terms with links to corresponding report pages. The user has the option of toggling between terms and genes and can follow links to ontology report pages for terms and to gene report pages for genes. Rounding out the diagram page are a list of references with links to the RGD reference report page (which links to PubMed), and a view of the ontology tree (Figure 4A-D). Pathways that are related, triggered by or directly connected to the featured pathway are shown in the diagram and they link to the ontology report for the term. In Figure 2 and 4A, the gray rectangles are pathway terms and they link to the corresponding ontology report pages, with links as described. If, as mentioned, a number of pathways revolve around a common concept or relate in a global fashion, they are presented in pathway suites and suite networks which offer an instant snapshot of their relatedness. For instance, the folate cycle and the folate mediated one-carbon pathways, the methionine, homocysteine and other metabolic pathways are components of the ‘Methionine, Homocysteine, Folate and Related Metabolites Pathway Suite’. The pathway suites dedicated to the pro- and the anti-inflammatory signaling pathways are the two arms of the ‘Balancing Inflammatory Responses Pathway Suite Network’ (see also the ‘Glucose Homeostasis Pathway Suite Network above). A tripartite pathway suite network dedicated to molecular mechanisms of blood pressure regulation has also recently been released. Thus, whether from within individual diagrams or via suites and suite networks, the user can travel the pathway landscape, from detailed examination to broad overview.

Primarily the review and research literature published in major journals, but also database searches and users requests, are sources for the addition of terms in the ontology, the representation of pathways in interactive diagrams and the annotation of genes within pathways. In addition, the restructuring of the drug node and pipelines for importing pathway data from external databases helped, and new disease portal releases continue to help increase and improve upon the content, structure and use of the ontology. These more recent developments are presented in the next section.

## Results and discussion

To further expand the information content that the Pathway Portal provides, RGD has recently developed automated pipelines to bring in data from external sources. The building of the pipelines, along with the ongoing development of the ontology and the deployment of disease portals, has led to further developments within the pathway ontology. Many of the new terms added for the pipelines are within the signaling and disease pathway nodes of the ontology. The restructuring of the drug pathway node was accompanied by the addition of new terms, particularly for the drugs within the antineoplastic category. User requests led to additions across the metabolic, signaling and regulatory pathway nodes. Since last presented [17], the ontology has seen the addition of more than 640 new terms across all nodes of the ontology, representing ~44% of the current ontology content. As of the time of this writing, the ontology holds over 1,480 terms (see Table 1 for ontology statistics).

**Table 1:** A summary of PW aspects and structure

**Number of class nodes**	**Relationship_types**	**Total number of terms**	**Total number of annotations**	**Depth**	**Definition coverage**
5	is_a; part_of	1,485	49,103	10	86%

### Pathway interaction database (PID) pipeline

The Pathway Interaction Database (PID) [21] at the National Cancer Institute has been offering a collection of human regulatory and signaling pathways and has been using the regulatory and signaling nodes of PW to allow its users to browse these pathways by categories. To load and access the manually curated human PID annotations, RGD decided to implement a pipeline that would automatically bring in the data. PID pathway identifiers (IDs) were added as synonyms in the Pathway Ontology. As PID was using higher level PW terms to categorize its pathways, names/terms in PID not present in PW were added. Building of the PID pipeline lead to the addition of several new terms within the signaling and regulatory nodes of PW. For instance, ‘ceramide signaling pathway’ and ‘sphingosine 1-phosphate signaling pathway’ were added to the ontology under ‘signaling pathway involving second messengers’ and a term for ‘lipid signaling pathway’ was added, as ceramide and sphingosine 1-phosphate are lipids. As mentioned, in the DAG structure, a child term can have more than one parent term. The two new terms are children of both ‘lipid signaling pathway’ and of ‘signaling pathway involving second messengers’. If more than one entry in PID was related to a term, all pertinent PID entry IDs were added as synonyms to PW. As an example, sphingosine 1-phosphate which acts intracellularly as a second messenger can also signal extracellularly as a ligand for several G protein coupled receptors. PID has separate entries for the receptors and they were added as synonyms to assure that all PID annotations pertinent to this lipid signaling were brought in. Other examples include terms for signaling by the members of the cadherin superfamily which increased the content under the ‘cell-cell signaling pathway’ parent term or those that increased the content under the ‘proteoglycan signaling pathway’ parent term. An accompanying literature search for the provision of definitions also lead to the addition of new terms. For instance, the PID entries for cadherin signaling are only for the E- and N-cadherins of the “classical” branch of the superfamily. Terms for the other branches of the cadherin superfamily were added at the same time.

The pipeline extracted the data from the PID master file and mapped it to PW terms via synonyms. 51 individual PW terms have synonyms that map to PID entries. The human genes in the PID file were matched to human genes in RGD and assigned to the mapped PW term with evidence code EXP (Inferred from Experiment). The annotations were propagated to the rat and mouse orthologs with the evidence code ISO (Inferred from Sequence Orthology). The use of evidence codes to indicate how the annotation of a gene to an ontology term is supported originates from the development of GO. Evidence codes are used for all ontology terms and objects that are annotated to them at RGD. Over 7,400 pathway gene annotations from PID are available at RGD (See Table 2 for number of mapping terms and annotations). In a gene report page, PID annotations are seen under the “Molecular Pathway Annotations” category with the source (PID) shown and also in the ‘External Database Link’ category under PID (and/or KEGG, as applicable, described below). The pathway(s) listed in the “Molecular Pathway Annotations” category link to the ontology report pages for those terms. The pathway(s) listed under the ‘External Database Link’ link to their entries at PID. Generally, RGD pipelines run on a weekly basis. Unfortunately, a few months after RGD released the pipeline, PID announced that it was no longer active and was retired in September 2013.

**Table 2:** A summary of term mappings and pathway annotations for the two pipelines

**Number of KEGG mappings**	**Number of KEGG annotations**	**Number of PID mappings**	**Number of PID annotations**
215	31,012	51	7,408

### The Kyoto encylopedia of genes and genomes (KEGG) pipeline

The Kyoto Encyclopedia of Genes and Genomes (KEGG) is a large and important pathway resource and provides a host of other biological information across a spectrum of phyla and species [22,23]. The KEGG Annotation Import Pipeline is based on a one-time download before the site changed its license and limited access to its data. KEGG map IDs were added as synonyms to the matching terms in PW and new ones were added, as necessary. For instance, KEGG has disease pathways by categories, including substance abuse and infectious diseases, which at the time were not represented in the ontology. These categories were added as parent terms with the corresponding children terms and the KEGG pathway map IDs as synonyms. Many individual child terms were added for the ‘infectious disease pathway’ category. KEGG and the literature were consulted to provide definitions that succinctly describe the condition and point to host pathway(s) and/or process(es) the condition may counteract or affect. Another new category was added for the ‘immune disease pathway’ with children terms for the entries at KEGG.

Pathway data was extracted from the KEGG master file and mapped to PW terms via synonyms. 215 PW terms have synonyms mapping to entries in KEGG. Genes from the KEGG annotations in the species files were matched to the RGD genes for rat, human and mouse and assigned to the corresponding PW term with evidence code IEA (Inferred from Electronic Annotation). Over 31,000 pathway gene annotations from KEGG are available at RGD. (See Table 2 for number of mapping terms and annotations). KEGG pathway annotations on RGD gene report pages are seen in a manner similar to the PID annotations.

### Drug pathway node – restructuring and applications

The drug pathway node was expanded to contain parent terms for categories as listed by the Anatomical Therapeutic Chemical (ATC) Classification System. The system is used for the classification of drugs and is controlled by the World Health Organization (WHO) Collaborating Centre for Drug Statistics Methodology [24,25]. In the ATC system, compounds are divided into groups depending on the organ or system upon which they act and their therapeutic, pharmacological and chemical characteristics. The main anatomical group represents the first level of the code. The second level of the code indicates the main therapeutic group while levels three to five indicate the therapeutic/pharmacological, the chemical/therapeutic/.pharmacological subgroups and the chemical substance, respectively. Initially, drug pathway terms were added directly under the main drug node. With the drug node expanding, the addition of terms for the first level categories as place holders for individual drug pathway terms was a necessary step to assure the consistency and internal logic of the ontology. In addition, a number of individual drug pathway terms were added, particularly for drugs within the ‘cardiovascular system drug pathway’ branch and for drugs in the ‘antineoplastic and immunomodulatory drug pathway’ branch. Many of the currently available disease pathway interactive diagrams represent cancer types and addition of diagrams for drug(s) used in cancer treatment allows linking the condition and the drug pathways. For each drug pathway term, children terms are added to represent the pharmacokinetics pathway (how the system processes the drug) and the pharmacodynamics pathway (how the drug acts upon the system). Examples include, but are not limited to ezetimibe, lomitapide and losartan drug pathways in the ‘cardiovascular system drug pathway’ branch, or axitinib, sunitinib, pazopanib, to name a few, in the ‘antineoplastic and immunomodulatory drug pathway’ branch. Of these, axitinib and losartan have interactive pathway diagrams currently available. As the node is expected to further expand including the development of new pipelines for data import from drug pathway databases, it is likely that the second level and/or other levels of ATC will be added as necessary in order to make both searching the tree and finding the pertinent entries easy while maintaining the consistency of the ontology. However, this and/or other restructuring involve branching off the tree and do not change the overall topology or architecture of the ontology. The ATC system can be browsed at KEGG [26].

As described, a disease pathway is represented by the altered pathways implicated in the disease process (see Figure 2). Various other elements are also provided on the disease pathway diagram including drugs and diseases, microRNAs whose expression is deregulated in tumor tissues, and other candidate genes, as applicable and/or available. As an example, in the case of renal cell carcinoma (RCC), alteration of the hypoxia inducible factor pathway is the major factor and several drugs are being used for treatment. Many of these drugs target the genes whose expression is controlled by hypoxia inducible transcription factors, such as components of the vascular endothelial growth factor (VEGF) pathway. Several drugs target the VEGF receptors; one such drug is axitinib. In the diagram page for this disease pathway, the icon for the drugs links to a page that lists them with links to the chemical entry pages (at RGD or elsewhere, as available) and to the available drug pathway(s). The icon for miRNA with potential roles in RCC and the one for other RCC candidate genes link to similar pages listing the pertinent entries. Such list pages are created in a Content Management System (CMS) and the url is added in the information for the object(s) created in the Ariadne Genomics Pathway Studio tool. The diagram for the axitinib drug pathway shows the overall actions of the drug along with side effects and links to associated pathway pages.

**Figure 5:**
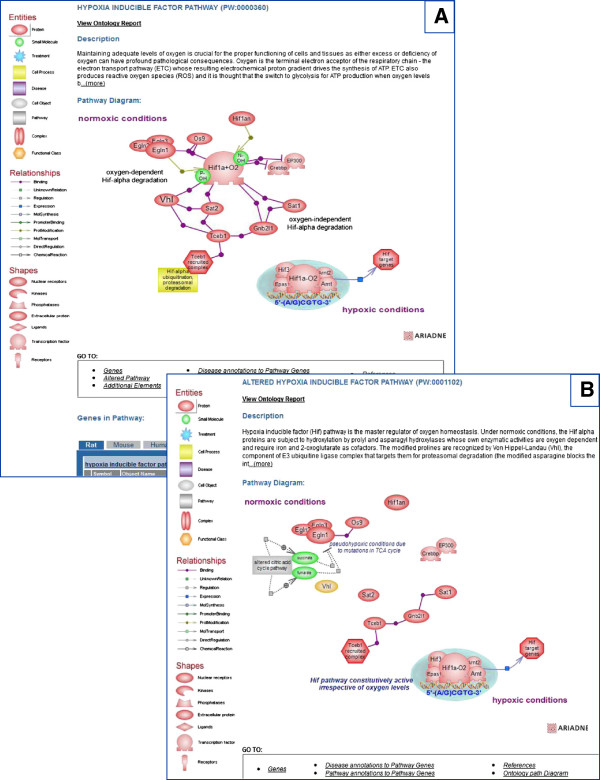
**Hypoxia inducible factor pathway. A.** The normal functioning of the ‘hypoxia inducible factor pathway’. **B.** The ‘altered’ version of the ‘hypoxia inducible factor pathway’.

In many instances, the pharmacokinetics of drugs is investigated in human liver microsomes. The microsomal enzymes belong to the families of the cytochromone P450 superfamily and are involved in drugs and xenobiotic metabolism. Some enzyme family members have broader substrate specificity, are less conserved and present considerable species as well as inter-individual variation [27,28]. In this case, annotations are not propagated to orthologs. For each altered version of a pathway, the normal pathway is built first. A pathway diagram page provides the option of adding the altered version of that pathway, if one exists. A direct link to the normal pathway is made available from the description on the altered pathway diagram page. Thus, users can see both the regular and the affected pathways, in this case the hypoxia inducible factor pathway and its altered version (Figure 5A, B). Culprit genes are color-coded in both the disease and the altered pathway diagrams (Figure 5B, see also Figure 2).

### Other developments

At the time the PID pipeline was built, RGD was in the process of developing the Immune and Inflammatory Disease Portal. As a result, the chemokine and the cytokine mediated signaling branches of the ontology were expanded to incorporate most if not all the chemokine and cytokine families or groups as parent terms with their corresponding members as children terms. The category ‘immune disease pathway’ was added as described in the KEGG pipeline section. More terms were added to cover the metabolism of vitamins. The deployment of new diagrams can also lead to increases in the content of and/or improvement in the consistency of the ontology. As an example, in the process of building the pathway diagrams to be included in the ‘Methionine, Homocysteine, Folate and Related Metabolites Pathway Suite’ both restructuring and additions have been made. In the transsulfuration pathway of homocysteine metabolism, hydrogen sulfide is a by-product of cysteine catabolism. While elevated levels can be toxic, the gaseous molecule can also act as a signaling molecule. ‘Hydrogen sulfide mediated signaling pathway’ and its parent ‘gasotransmitter mediated signaling pathway’ were therefore added to the ontology. Altered terms for the children of folate metabolism were also added, as several conditions resulting from defects in these pathways have been documented.

The development of the latest deployed disease portal – the Renal Disease Portal also contributed to the addition of terms across disease and drugs branches, among others. For instance, the above mentioned RCC pathway and the drugs that target it, such as axitinib, and the terms for the altered versions of the hypoxia inducible factor and the citric acid pathways, are examples of terms added for this portal. Currently, a portal for sensory organ diseases is under development that is expected to further contribute to the development of the ontology.

Users of the Pathway Ontology can contact RGD for requests of new terms and/or questions they might have. A recent request originally made for one pathway term has expanded into a large request list that further contributed to the increase in the content of the ontology within the metabolic, signaling and regulatory nodes. Children terms to parent entries within the carbohydrate, lipid and secondary metabolite branches of the metabolic node were added. The ‘metal’ and ‘non-metal ion transport pathway’ terms were created as children of the new parent term ‘ion transport pathway’ within the regulatory node, to accommodate the request for ‘chloride transport pathway’, a non-metal. Terms for several peptide hormone, growth and transcription factor signaling pathways were added within the respective parent terms in the signaling node. As of the time of this writing, other requests have been made with the addition of new terms in progress. The Pathway Ontology is species independent and terms for pathways that take place in species other than mammal or animal can be found. For instance, the pathways of secondary metabolites – a category within the metabolic node, are mostly present in microorganisms and/or plants. Likewise, the biodegradation of xenobiotics – also a category within the metabolic node, is the realm of microorganisms possessing the enzymes that are capable of breaking down chemicals generally resistant to degradation. Several user requests were for metabolic pathway terms present in plants and microorganisms. For instance, the biosynthesis of momilactone – a diterpene produced by rice, of cellulose – a polysaccharide and structural component of green plants cell walls, of lycopene – a carotene found in fruit and vegetables, or of a number of toxic secondary metabolites such as fumonisin and deoxynivalenol, were amongst those requests, to name a few.

The addition of new terms did not affect the overall structure of the ontology but helped increase the scope and coverage of pathway data that the users can access. In the course of expanding the content of the ontology, care was taken to appropriately assign terms to the parent or parents to which they belong and to provide adequate definitions. If necessary, new parent terms were created. Information available at the originating databases and that found in the literature were instrumental in the process.

## Conclusions

The Pathway Portal is an important project at RGD, with the Pathway Ontology providing the means for both the standardized annotation of rat, human and mouse genes and for easy navigation between the components of the portal and from various entry points. The navigational aspect is an important one, given the extent of the pathway data coverage RGD offers. The recent addition of data import pipelines has helped to further expand the content of PW and the pathway data RGD provides. Additional pipelines are envisioned to be built in the near future. Literature survey continues to be important for the ongoing development of the Pathway Ontology and Portal. User requests and external projects using PW are additional sources for expanding the content of the ontology and for enhancing its structure. A primary goal of the Pathway Ontology is to capture the pathway/network universe and its attributes and to articulate the connections and relationships between them within a hierarchical structure. This includes capturing the malfunctioning of the system and the attempts to restore it. The five nodes of the ontology along with the provision of altered pathways address this goal. The branching of the tree, whether branching within a node or expanding within a class, does not affect the overall topology and architecture of the ontology. The literature, external databases and resources, and expert opinions internally and externally help assure that the provision of new terms represents the current knowledge and understanding of biological events and are reflected as such within the ontology. Table 1 summarizes the structure of the ontology; Table 2 summarizes the term mappings and pathway annotations of the two pipelines.

## Methods

The Pathway Ontology (PW) is being built using the OBO-Edit ontology editor, a freely available Java-based tool developed and maintained by the GO Consortium [3,29]. Updated versions of the ontology are uploaded into the database, placed in the RGD ftp site and uploaded to the NCBO BioPortal PW site (see Abstract, [30,31]). For the deployment of pipelines, the identifiers (IDs) used by the external databases are added as synonyms to their corresponding PW terms, in the OBO-Edit tool.

The pathway diagrams are being built using the Ariadne Genomics Pathway Studio version 8, originally from Ariadne Genomics and currently available at Elsevier [32]. The tool comes with the mammalian ResNet database which contains a large set of objects such as genes, diseases, treatments, chemicals and others along with their accompanying information. In addition, ResNet allows for new properties to be added and values to be attributed to them. These features have been exploited to add PW and other IDs, as well as urls for chemicals and for pages containing lists of objects that are accessible from the diagram pages. These pages are created using a Content Management System (CMS) – a computer program for publishing, editing and modifying content for easier management of workflow. The diagrams, saved as HTML, are folders containing the diagram file and files for every object present in the diagram with the information that object has in the ResNet database. A script parses these files for PW:IDs, RGD:IDs and url links that have been added, rendering these objects linkable to the corresponding sites. The diagram pages are being created using a web application developed at RGD [18].

The KEGG and PID pipelines are Java 1.6 standalone applications. The pipelines communicate with an Oracle database, the RGD database, using the Oracle JDBC thin client driver. The Spring framework v.1.2 is used for dependency injection, and parameters are loaded from an external file allowing for easy pipeline customization [33]. Apache commons libraries are used for database connection pooling, and Log4j provides extensive logging capabilities [34]. Database access objects from the RGD framework provide a stable data model giving the pipeline developer a thoroughly tested and efficient API for accessing the RGD database. The pipeline code is stored in the RGD subversion code repository. Once a pipeline is ready to move to production, a job is created and configured within the Hudson Continuous Integration Server allowing the pipeline to be rebuilt or deployed directly from source.

## Abbreviations

API: Application programming interface; ATC: Anatomical therapeutic chemical classification system; CMS: Content management system; DAG: Directed acyclic graph; EXP: “Inferred from experiment” evidence code; GO: Gene ontology; IEA: “Inferred from electronic annotation” evidence code; ISO: “Inferred from sequence orthology” evidence code; JDBC: Java database connectivity; KEGG: The kyoto encyclopedia of genes and genomes; miRNA: Microrna; NCBO: National center for biological ontology; PID: Pathway interaction database; PNG: Portable network graphics file format; RCC: Renal cell carcinoma; RGD: Rat genome database; SMPDB: Small molecule pathway database; WHO: World health organization.

## Competing interests

The authors declare that they have no competing interests.

## Authors’ contributions

VP wrote the manuscript, originated the PW ontology, composed most of the PW diagrams, assisted with portal design, diagram publications and with pathway pipeline construction. PJ assisted with construction of pathway pipelines. MT assisted with PW uploads, ftp file placement and reviewed manuscript. GTH composed some of the PW diagrams, assisted with diagram publications and reviewed manuscript. JRS assisted with diagram publications. JDP supervised pipeline construction. TFL reviewed manuscript. RN is part of the RGD team. SJFL reviewed manuscript. SJW reviewed manuscript. MS supervised PW portal and pipeline projects. MRD, DHM, EAW, HJJ co-supervised RGD projects. All authors read and approved the final manuscript.
